# Transcriptomic response of lumpfish (*Cyclopterus lumpus*) head kidney to viral mimic, with a focus on the *interferon regulatory factor* family

**DOI:** 10.3389/fimmu.2024.1439465

**Published:** 2024-08-15

**Authors:** Mohamed Emam, Surendra Kumar, Khalil Eslamloo, Albert Caballero-Solares, Jennifer R. Hall, Xi Xue, Hélène Paradis, Robert L. Gendron, Javier Santander, Matthew L. Rise

**Affiliations:** ^1^ Department of Ocean Sciences, Memorial University of Newfoundland, St. John’s, NL, Canada; ^2^ Centre for Marine Applied Research, Dartmouth, NS, Canada; ^3^ Aquatic Research Cluster, Core Research Equipment and Instrument Training (CREAIT) Network, Ocean Sciences Centre, Memorial University of Newfoundland, St. John’s, NL, Canada; ^4^ Faculty of Medicine, Memorial University of Newfoundland, St. John’s, NL, Canada; ^5^ Marine Microbial Pathogenesis and Vaccinology Laboratory, Department of Ocean Sciences, Memorial University of Newfoundland, St. John’s, NL, Canada

**Keywords:** antiviral, lumpfish (*Cyclopterus lumpus*), IRF, RNA seq, qPCR

## Abstract

The economic importance of lumpfish (*Cyclopterus lumpus*) is increasing, but several aspects of its immune responses are not well understood. To discover genes and mechanisms involved in the lumpfish antiviral response, fish were intraperitoneally injected with either the viral mimic polyinosinic:polycytidylic acid [poly(I:C)] or phosphate-buffered saline (PBS; vehicle control), and head kidneys were sampled 24 hours post-injection (hpi) for transcriptomic analyses. RNA sequencing (RNA-Seq) (adjusted p-value <0.05) identified 4,499 upregulated and 3,952 downregulated transcripts in the poly(I:C)-injected fish compared to the PBS-injected fish. Eighteen genes identified as differentially expressed by RNA-Seq were included in a qPCR study that confirmed the upregulation of genes encoding proteins with antiviral immune response functions (e.g., *rsad2*) and the downregulation of genes (e.g., *jarid2b*) with potential cellular process functions. In addition, transcript expression levels of 12 members of the interferon regulatory factor (IRF) family [seven of which were identified as poly(I:C)-responsive in this RNA-Seq study] were analyzed using qPCR. Levels of *irf1a*, *irf1b*, *irf2*, *irf3*, *irf4b*, *irf7*, *irf8*, *irf9*, and *irf10* were significantly higher and levels of *irf4a* and *irf5* were significantly lower in the poly(I:C)-injected fish compared to the PBS-injected fish. This research and associated new genomic resources enhance our understanding of the genes and molecular mechanisms underlying the lumpfish response to viral mimic stimulation and help identify possible therapeutic targets and biomarkers for viral infections in this species.

## Introduction

Lumpfish (*Cyclopterus lumpus*) are commonly used as an environmentally friendly solution for sea lice control (e.g., *Lepeophtheirus salmonis*) in Atlantic salmon (*Salmo salar*) farms in the North Atlantic region (1–3). Sea lice infestations lead to decreased fish health, growth, and, consequently, market value (4). Additionally, lumpfish as a biological method to control sea lice help reduce the reliance on chemical treatments, which contribute to environmental pollution. However, lumpfish farming faces several challenges. For example, lumpfish are susceptible to several infectious diseases, which can be transferred to other aquatic hosts such as Atlantic salmon (2, 5). Atlantic salmon and lumpfish can both be infected with pathogens such as the bacterium *Renibacterium salmoninarum* and the viral hemorrhagic septicemia (VHS) virus (2, 6). While lumpfish may be infected by viral pathogens (e.g., **Supplementary Table S1**), the development of vaccines for farmed lumpfish has thus far focused on bacterial pathogens (7–12) rather than viruses. The development of vaccines for the protection of lumpfish against viral infection is a priority (7).

Polyinosinic:polycytidylic acid [poly(I:C)] is a synthetic analog of double-stranded RNA (dsRNA) that can mimic viral infections (i.e., elicit a potent antiviral-like response) in several species including teleosts and is commonly used as an immune stimulant in aquaculture research (13–15). Poly(I:C) was previously used in several studies to evaluate the antiviral response of zebrafish (*Danio rerio*), Chinook salmon (*Oncorhynchus tshawytscha*), seven-band grouper (*Epinephelus septemfasciatus*), and Atlantic salmon, as it mimics RNA viral pathogens of fish (15–19). For example, positive-sense single-stranded RNA (ssRNA) viruses such as nodaviruses and flaviviruses form dsRNA intermediates during their replication cycle (20–22). Also, it has been reported that lumpfish can be infected with dsRNA viruses (**Supplementary Table S1**), such as *C. lumpus* toti-like virus (CLuTLV) (23), and ssRNA viruses that likely produce dsRNA during their replication cycle including *C. lumpus* virus (CLuV) (24) and nervous necrosis virus (NNV) (25).

RNA sequencing (RNA-Seq) is a highly robust method for assessing global gene expression responses, as it generates accurate and reproducible results (26). Transcriptomic studies may be used to identify genes and pathways that respond to immune challenges. As examples, the transcriptomic responses to viral infection [e.g., infectious salmon anemia virus (ISAV) and infectious pancreatic necrosis virus (IPNV)] and/or poly(I:C) were previously explored in Atlantic salmon, rainbow trout (*Oncorhynchus mykiss*) (27), red-spotted grouper (*Epinephelus akaara*) (28), yellowhead catfish (*Tachysurus fulvidraco*) (29), ya-fish (*Schizothorax prenanti*) (30), and yellow catfish (*Pelteobagrus fulvidraco*) (31). To our knowledge, the lumpfish transcriptomic response to a viral pathogen has not been studied. While lumpfish primary leukocytes’ transcript expression responses to poly(I:C) have been studied recently (32, 33), the lumpfish systemic immune antiviral transcriptomic response [e.g., response of immune tissue/organ such as head kidney to *in vivo* stimulation with poly(I:C)] had not been characterized prior to the current study. Characterization of the lumpfish head kidney transcriptomic response to poly(I:C) will provide a foundation for understanding the mechanisms underlying the lumpfish immune response to viral infections, thereby aiding in the development of vaccines and the improvement of aquaculture practices.

In addition to the transcriptomic response to poly(I:C), several aspects of lumpfish antiviral mechanisms remain uncharacterized. Interferon regulatory factor (IRF) family members are key elements of fish immune responses (34). Transcript expression levels of several members of the IRF family (e.g., IRF3, 5, and 7) are upregulated following viral infections (34). IRFs are transcription factors, and their activation leads to the induced expression of interferons (IFNs) and IFN-stimulated genes (ISGs), which play crucial roles in antiviral responses. Additionally, *irf* expression is dysregulated in response to several stressors such as viral and bacterial infections, heat shock, and toxins (35–37). Moreover, while members of the IRF family are suggested to be highly conserved in their structure across vertebrate species, they also play some species-dependent roles (38–40). Several members of the IRF family were characterized in teleost species such as common carp (*Cyprinus carpio* L.) (41), Japanese flounder (*Paralichthys olivaceus*) (42), turbot (*Scophthalmus maximus*) (43, 44), Japanese seabass (*Lateolabrax japonicus*) and Atlantic cod (*Gadus morhua*) (45–47). However, the antiviral response of the lumpfish *irf* family members remained unknown prior to this study. In addition to transcriptomic profiling, in this study, we focused on evolutionary aspects and poly(I:C) responses of *irf* family members to investigate if these genes play conserved roles in lumpfish.

In the current study, we analyzed the transcriptomic response of lumpfish head kidney to intraperitoneal (IP) injection with poly(I:C) using RNA-Seq. Real-time quantitative polymerase chain reaction (qPCR) analyses were then utilized to assess expression levels of selected transcripts (e.g., representing hub genes in pathway analyses and well-known antiviral biomarkers) to confirm the results of the RNA-Seq analyses and, specifically, to elucidate the expression profiles of the 12 members of the lumpfish *irf* family in response to poly(I:C). Also, we investigated the molecular phylogeny of IRF members from lumpfish and three other teleost species representing different superorders to improve our understanding of the evolutionary history of the IRF family across Teleostei. The results of the current study enhance our knowledge of the genes and molecular pathways involved in the antiviral immune responses of fishes.

## Materials and methods

### Animals, experimental design, and sample collection

Juvenile lumpfish were raised at the Dr. Joe Brown Aquatic Research Building (JBARB), Ocean Sciences Centre, Memorial University of Newfoundland, Canada (48). At 300 days post-hatch, 18 fish (average weight ± standard deviation = 85.4 ± 14.6 g) were randomly selected and distributed into three 500-L tanks. The fish were held in 8–10°C filtered and UV-treated seawater, with a flow rate of 7.5 L/min, and the oxygen level was maintained at a saturation range of 95%–110%. The photoperiod was 12-h light and 12-h dark. The fish were fed a commercial diet (Marine grower diet, Zeigler Bros., Inc., Gardners, PA, USA) at 0.5% of the average body weight per day.

**Figure 1:**
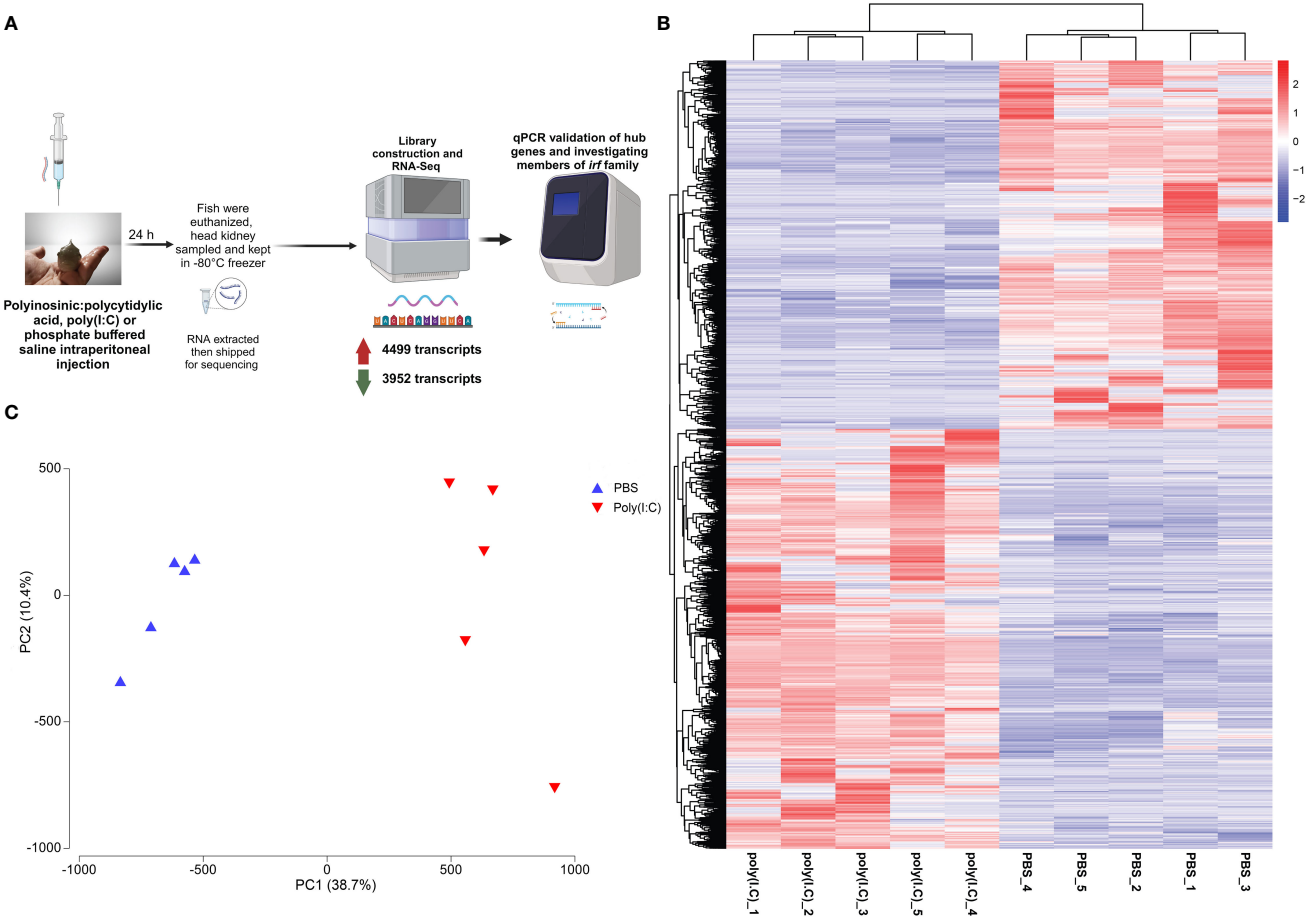
**(A)** Overview of the methods used in the current study. This figure was generated using Biorender.com. **(B)** Heatmap and hierarchical clustering of polyinosinic:polycytidylic acid [poly(I:C)]-responsive transcripts [i.e., differentially expressed transcripts (DETs)]. The transcripts per million (TPM; log_2_ transformed) values for each DET are clustered along the vertical axis, the ID of the sample subjected to RNA-Seq is shown on the base of the figure, and the sample cluster is plotted on the top of the map. **(C)** Principal component analysis of DET of all the samples used in sequencing using TPMs.

After 1 month, the poly(I:C) challenge study was conducted. Briefly, the fish were fasted for 24 h and then lightly anesthetized with MS-222 (50 mg/L, Syndel Laboratories, Vancouver, BC, Canada). Three individuals per tank were intraperitoneally injected with either poly(I:C) [2 µg/g fish, dissolved in phosphate-buffered saline (PBS)] or PBS (sham-injection/vehicle control). Immediately after injection, each fish was stitched with nylon surgical thread on one fin [the left pectoral fin for poly(I:C) or the right pectoral fin for PBS] and then returned to the same tank. At 24 hours post-injection (hpi), fish were euthanized using MS-222 (400 mg/L), and the head kidneys were collected. The samples were flash-frozen in liquid nitrogen and stored at −80°C until RNA extraction. All procedures in this experiment were performed following the Canadian Council of Animal Care guidelines (Memorial University of Newfoundland Animal Care Protocol, 17–03-RG and 18–01-MR) and in accordance with ARRIVE guidelines (https://arriveguidelines.org). **Figure 1A** depicts an overview of the workflow in this study.

### Total RNA sample preparation

The RNA extractions were performed as described in Emam et al. (49). Briefly, the head kidney samples were homogenized in TRIzol Reagent (Invitrogen/Thermo Fisher Scientific, Burlington, ON, Canada) using stainless steel beads (5 mm; QIAGEN, Mississauga, ON, Canada) and a TissueLyser (QIAGEN), and the total RNA extractions were then completed following the manufacturer’s instructions. Total RNA samples (~25 µg) were treated with 6.8 Kunitz units of DNaseI (RNase-Free DNase Set, QIAGEN) for 10 min at room temperature and then purified using the RNeasy MinElute Cleanup kit (QIAGEN) following the manufacturer’s instructions. The integrity and the purity of the purified RNA were evaluated using 1% agarose gel electrophoresis and NanoDrop spectrophotometry (NSW-1000), respectively. The RNA samples used in this study showed high integrity (i.e., tight 28S and 18S ribosomal RNA bands at a ratio of ~2:1) and purity (i.e., A260/280 and A260/230 ratios above 1.9 and 2.0, respectively).

### RNA−seq analysis

Library construction and RNA-Seq services were performed at the Centre d’expertise et de services (CES), Génome Québec, Montréal, QC, Canada. Prior to library construction, RNA quality was further evaluated using the Bioanalyzer 2100 (Agilent, Santa Clara, CA, USA), and all samples were of high integrity (i.e., with RNA Integrity Numbers of 9.8–10). Ten libraries (i.e., for head kidney RNA samples from five of the nine poly(I:C)-injected fish and five of the nine PBS-injected fish) were constructed using the NEBNext mRNA Library Prep Reagent Set for Illumina (New England Biolabs, Whitby, ON, Canada) following the manufacturer’s instructions. Sequencing was performed on the Illumina NovaSeq 6000 [S2] with paired-end 100 bp (PE100) and at least 50 million reads per library. A summary of the RNA-Seq and library construction can be found in **Supplementary Table S2**.

### RNA-seq data processing

The quality control, trimming, and filtering of low-quality reads were performed using FastQC version 0.11.8 and Trimmomatic version 0.3971. The sequencing reads were aligned to the lumpfish genome [*C. lumpus* (assembly fCycLum1.pri), ID: 86363] using HISAT2 version 2.1.0. StringTie version 2.0 was used to assemble and calculate the expression levels of all transcripts using reference gene models provided in the form of Gene Transfer Format (GTF) annotation files that are distributed with the lumpfish genome. StringTie was also used to assemble and quantify novel genes and transcripts. The accuracy of transcript assembly was evaluated using gffcompare version 0.11.2. The read count data used for differential expression analysis were obtained from the python script “prepDE.py” provided by the StringTie authors. The differential expression analysis was performed using DESeq2 version 1.28.1 in the Bioconductor package at an adjusted p-value (padj) threshold of <0.05, |log_2_ fold change| > 1, and filtering for transcripts with expression transcripts per million (TPMs) greater than 1 in at least two replicates in each group.

**Figure 2:**
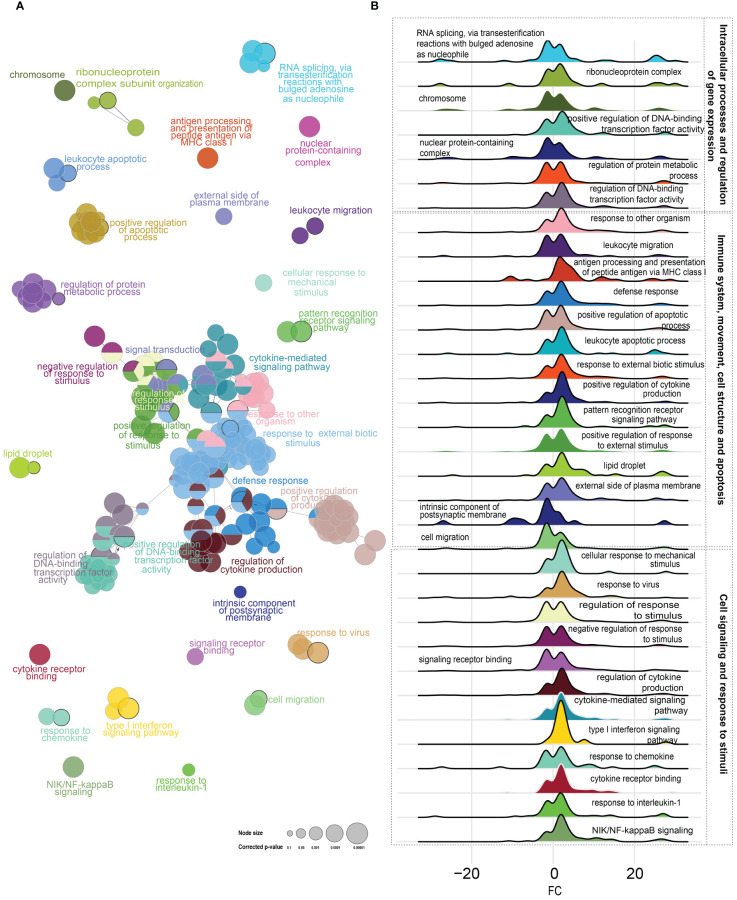
**(A)** Gene Ontology (GO) term enrichment and pathway term network analysis of differentially expressed transcripts (DETs). The GO term enrichment analysis was performed using ClueGO plugin in Cytoscape. The p-value was adjusted at 0.05, kappa score level was ≥0.4 on ClueGO, and Benjamini–Hochberg correction was used. Biological process, cellular component, and molecular function were the selected ontologies on ClueGO. Nodes represent enriched GO terms. A complete list of the enriched GO terms is found in **Supplementary Table S4**, while the leading GO terms are also labeled in the figure. **(B)** Density plots of the fold change for the leading GO terms, showing the upregulated and downregulated genes in each GO term.

A heatmap with the TPM values (log_2_ transformed) of all differentially expressed transcripts (DETs) was generated using the “heatmap3” function of the “gplots” package in R (2023.03.1; **Figure 1B**). A principal component analysis (PCA; **Figure 1C**) was performed using the standardized TPMs of all of the identified DETs. A volcano plot was used to show significance (−log_10_ padj) against the expression log_2_ fold change (LFC). Gene Ontology (GO) term enrichment analysis was conducted using the ClueGO (50) plugin of Cytoscape (51) version 3.5 and using the *C. lumpus* ontology database. The enrichment analyses considered biological process, cellular component, and molecular function. The distribution of up- and downregulated transcripts within each leading GO term were plotted by LFC using density plots (**Figure 2B**). Leading GO terms were then manually classified based on related function into 1) intracellular processes and regulation of gene expression; 2) immune system, movement, cell structure, and apoptosis; and 3) cell signaling and response to stimuli (**Figure 2**). A circular bar plot was used to show GO terms with the highest percentage of DETs, after removing redundant GO terms, using library(tidyverse). The standard lumpfish gene symbols for all transcripts in our RNA-Seq dataset were used as references for the enrichment analyses, which were performed at the transcript level since ClueGO conducts the analyses using standard symbols. The heatmap and hierarchical clustering of DETs (using TPMs; log_2_ transformed) that participated in enriching “response to virus”, “regulation of retinoic acid receptor signaling pathway”, and “response to lipid” were generated using TBtools software (52).

### qPCR overview

To confirm the results of the RNA-Seq analyses, transcript expression levels of 18 genes (either hub genes or known antiviral biomarkers) that were identified as differentially expressed were also assessed using qPCR. In addition, expression levels of the 12 members of the lumpfish *irf* family [seven of which were also identified as poly(I:C)-responsive by RNA-Seq] were assessed to elucidate the response of this gene family to poly(I:C). Levels were assessed in all of the 18 study samples (i.e., head kidney samples from the poly(I:C)- and PBS-injected fish at 24 hpi; n = 9 per group).

### cDNA synthesis and qPCR parameters

First-strand cDNA templates for qPCR were synthesized in 20-µL reactions from 1 µg of DNaseI-treated, column-purified total RNA using random primers (250 ng; Invitrogen), dNTPs (0.5 mM final concentration; Invitrogen), and M-MLV reverse transcriptase (200 U; Invitrogen) with the manufacturer’s first-strand buffer (1× final concentration) and DTT (10 mM final concentration) at 37°C for 50 min.

The qPCR amplifications were performed in 13 µL reactions containing 1× Power SYBR Green PCR Master Mix (Applied Biosystems, Foster City, CA, USA), 50 nM of both the forward and reverse primers, and the indicated cDNA quantity. All reactions were performed in triplicate and included no-template controls (NTCs). Amplifications were performed using either the ViiA 7 Real-Time PCR system or the QuantStudio 6 Flex Real-Time PCR system (384-well format) (Applied Biosystems). The qPCR analysis program included 1 cycle of 50°C for 2 min, 1 cycle of 95°C for 10 min, and 40 cycles of 95°C for 15 s and 60°C for 1 min, with fluorescence detection at the end of each 60°C step, and was followed by dissociation curve analysis.

### Primer design and quality assurance testing

Previously published (2) and newly designed qPCR primers were used in this study. For the 25 qPCR-analyzed genes that were identified as differentially expressed in the RNA-Seq analyses, a BLASTn search of the non-redundant nucleotide (nr/nt) sequence database of the National Center for Biotechnology Information (NCBI) [*C. lumpus* (taxid: 8103) sequences only] was performed to identify annotated sequences corresponding to the transcript sequence generated herein [i.e., transcript of interest (TOI)]. This search also determined if gene paralogues/isoforms were present; the additional five *irf* family members were identified using BLASTn. A database of the sequences obtained from GenBank was compiled using Vector NTI (Vector NTI Advance 11.5.4, Life Technologies, Carlsbad, CA, USA). For a given gene, if paralogues/isoforms were present, multiple sequence alignments were performed using AlignX (Vector NTI Advance 11.5.4) to identify regions where paralogue/isoform-specific qPCR primers for the TOI could be designed (i.e., in an area with ≥3-bp difference between them). However, if sequences for transcript variants were present, primers were designed in a region that was conserved among the variants and generated identical amplicons. Most primers were designed using either PrimerQuest (www.idtdna.com/Primerquest/Home/Index) or Primer3 (53, 54) however, some were manually designed in paralogue/isoform-specific areas to ensure specificity. All primers had a melting temperature (T_m_) of 60°C and were located in the CDS.

The qPCR primers utilized herein were subjected to quality control testing as described previously (55). All showed single-product amplification and the absence of primer dimer in the NTC using dissociation curve analysis. Amplification efficiencies were calculated for two cDNA pools generated from all individuals in the PBS group and from all individuals in the poly(I:C) group. Standard curves were generated using a 5-point 1:3-fold dilution series starting with cDNA representing 10 ng of input total RNA. The reported efficiencies are an average of the two values. The sequences, amplicon sizes, and efficiencies for all primer pairs used in the qPCR analyses are presented in **Table 1**.

**Table 1:** Primers used in qPCR analysis.

Gene name	Symbol	GenBank accession number	^a^E (%)	^b^Nucleotide sequence (5′–3′)	Amplicon size (bp)	Reference
*Activating transcription factor 3*	*atf3*	XM_034528323	97.6	F:AGGAGCTGAAGCAGCAGAAG	135	This study
				R:TGCTCTCCTTGATGTGTTGC		
*ADAM metallopeptidase domain 22*	*adam22*	XM_034553371	96.8	F:CCAGTGTCCAACAAATGTGC	143	This study
				R:AGAACTTGTCAGCCGCTGTT		
*ADAM metallopeptidase with thrombospondin type 1 motif, 15a*	*adamts15a*	XM_034550916	92.9	F:GACCAGCCTCAGAAACCGTT	120	This study
				R:TGGCTGCATAAAGGGACAGG		
*ATP-dependent RNA helicase lgp2*	*dhx58* (*lgp2*)	XM_034539875.1	95.2	F:GCAACCTGGTGGTACGCTAT	104	(2)
				R:CTCGGCGACCACTGAATACT		
*Adenosine monophosphate deaminase 2b*	*ampd2b*	XM_034537011	80.6	F:CACGTTGTGGGTTTTGACAG	100	This study
				R:TGTGCTCCTCTGTCCAGTTG		
*Cholesterol 25-hydroxylase like 3*	*ch25hl3*	XM_034540810	89.9	F:GCTCTCTGGAGCTGCTGTCT	103	This study
				R:CAGCTGTTGATGAGGTGGAA		
*E3 ubiquitin/ISG15 ligase TRIM25-like*	*trim25*	XM_034531793	91.8	F:CTCCTTCCTCTGTGTGTTTATGG	80	This study
				R:TCCTGCAGATGAATATGAGTTCAG		
*Interferon-induced GTP-binding protein Mx-like*	*mx1*	XM_034531951	90	F:TGCACAGACTCAAGCAGAGC	144	(2)
				R:CCACACTTGAGCTCCTCTCC		
*Interferon alpha/beta receptor 2-like*	*ifnar2*	XM_034560853	90.6	F:ACATGGAGCACACACTGAGC	80	This study
				R:CGGCTGTCAGTTTCAAACAA		
*Interleukin-1 beta-like*	*il1b*	XM_034542525	104	F:ATTGTGTTCGAGCTCGGTTC	98	(2)
				R:CGAACTATGGTCCGCTTCTC		
*Jumonji, AT rich interactive domain 2b*	*jarid2b*	XM_034544699	100	F:CTGGTGTACTTGGATGCGGT	111	This study
				R:AAAACGCATCTCCTCGCTCA		
*PHD finger protein 8*	*phf8*	XM_034538118	96.4	F:AGTAATGGTGCAGGAAGGGC	103	This study
				R:GGGTTTCGTCAATCTGCAGC		
*Radical S-adenosyl methionine domain containing 2*	*rsad2*	XM_034563028	92	F:AGGAGAGGGTGAAGGGAGAG	133	(2)
				R:ATCCAGAGGCAGGACAAATG		
*Sacsin*	*sacs*	XM_034549198	82.9	F:CCAGATTGGTACTGCCTGGT	102	This study
				R:GTCCGAGTTGTCCATGTGTG		
*Sacsin-like*	*sacs-like*	XM_034562115	90.3	F:CAGACGATGCTAAAGCCACA	111	This study
				R:CGTAGAGAGCAGGACCTTGG		
*Toll-like receptor 7*	*tlr7*	XM_034560839	92.1	F:GGCAAACTGGAAGAATTGGA	100	(2)
				R:GAAGGGATTTGAGGGAGGAG		
*Tripartite motif-containing protein 16-like*	*trim16*	XM_034532965.1	97.9	F:GGAGTCGACTAAACATCCAGCA	209	This study
				R:TCGACTCACTTCAGTTCTCTGC		
*Vesicle-associated membrane protein 8* (*endobrevin*)	*vamp8*	XM_034541982	92.8	F:GGTGGCTGGAGTGAAAGACA	144	This study
				R:CGAGCCACTTTCTGAGACGT		
*^c^Interferon regulatory factor 1a*	*irf1a*	XM_034527913	89.6	F:CAAGCCAGATCCCAAGACAT	100	This study
				R:GCTGCCTCTCTTCTTGCTGT		
*Interferon regulatory factor 1b*	*irf1b*	XM_034551153	101	F:CCGGCTTCTCAAACAACTTC	112	This study
				R:GAGTCTTTCTCCGGTTGCTG		
*Interferon regulatory factor 2*	*irf2*	XM_034543211	101	F:GCTTCCCACGTGTCCTCTAC	110	This study
				R:CGGTGTGGTAGCTGATGAGA		
*Interferon regulatory factor 3*	*irf3*	XM_034559314	91	F:TCATTGAGGGGAGAAACTGC	118	This study
				R:GTCAGGACCACCTCCACTGT		
*Interferon regulatory factor 4a*	*irf4a*	XM_034554744	97.5	F:TCAGGAGAGAAGGGACTGGA	120	This study
				R:AACGGTGACGGATAGTGGAG		
*Interferon regulatory factor 4b*	*irf4b*	XM_034529934	97.5	F:CAGGGAGGACTGTCCCAGTA	108	This study
				R:CCCGTAGCTCTGGATTTCTG		
*Interferon regulatory factor 5*	*irf5*	XM_034526472	97.5	F:GTCCAGGTTGTTCCTGTCGT	130	This study
				R:TGAACTGCTCCACTGTCTGG		
*Interferon regulatory factor 6*	*irf6*	XM_034533756	110	F:CTTCGGGCCAGTGAACTTAG	125	This study
				R:AGGCCTCTGTCCATCACATC		
*Interferon regulatory factor 7*	*irf7*	XM_034535915	104	F:GAATTCGGACGACCCTCATA	140	This study
				R:CTGAGGGGAAGCACTCTACG		
*Interferon regulatory factor 8*	*irf8*	XM_034528338	99.9	F:CAGCCCTGCAGAGATAGAGG	109	This study
				R:CCTGATGCAGATGAAAAGCA		
*Interferon regulatory factor 9*	*irf9*	XM_034560118	84.9	F:AGTTCACGGAGGTGATGGAG	119	This study
				R:CTTCGCTCTGGGCTTCTTCT		
*Interferon regulatory factor 10*	*irf10*	XM_034537944	100	F:TGATCCAGGCTCTGAGGTCT	111	This study
				R:CATCGGGCAACGTCTTTACT		
*60S ribosomal protein L32*	*rpl32*	XP_034392188.1	100	F:GTAAGCCCAGGGGTATCGAC	107	(2)
				R:GGGCAGCATGTACTTGGTCT		
*Elongation factor 1-alpha*	*ef1a2a*	XM_034545962.1	98.1	F:GAGAAGATGGGCTGGTTCAAG	87	This study
				R:GGCATCCAGAGCCTCCA		

a E, efficiency.

b F, forward; R, reverse.

c Both *irf1a* (named on NCBI as *irf1-like*) and *irf4b* (named on NCBI as *irf4-like*) were re-named based on that of the closest orthologues in the phylogenetic tree (**Figure 3**).

**Figure 3:**
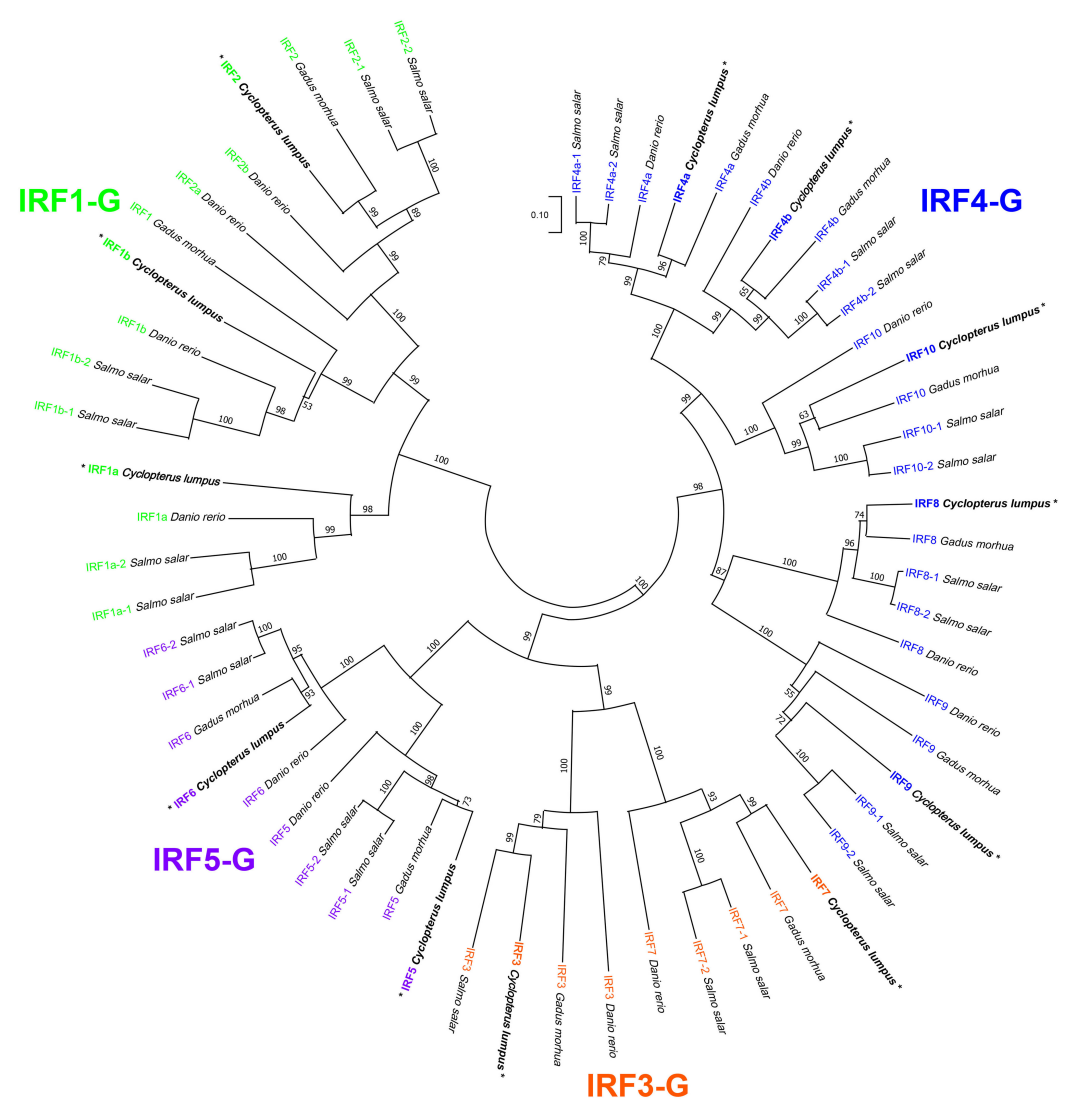
Phylogenetic tree analysis of putative interferon regulatory factor (IRF) orthologues across the four teleost superorders. Putative IRF amino acid sequences from a fish representing each of the four teleost superorders, namely, Protacanthopterygii [*Salmo salar* (Atlantic salmon)], Acanthopterygii [*Cyclopterus lumpus* (lumpfish)], Paracanthopterygii [*Gadus morhua* (Atlantic cod)], and Ostariophysi [*Danio rerio* (zebrafish)] were collected from the National Center for Biotechnology Information (NCBI) non-redundant protein database (see **Supplementary Table S5**). The 59 amino acid sequences were aligned using ClustalW, and the tree was constructed using the Neighbor-Joining method with Poisson correction; the bootstrap test of phylogeny was performed with 10,000 replicates in the MEGA 11 (v.11.0.13) (60) software. The numbers at the branch points represent the bootstrap values, and branch lengths are proportional to calculated evolutionary distances. The scale represents the number of amino acid substitutions per site. The Atlantic salmon IRF paralogues were named as suggested in Clark et al. (2021), with the exceptions being IRF2–1 and IRF2–2, which were named as in Crossman et al. (2023); IRF1a (alias IRF11). The four subgroups—IRF1-G (IRF1 and IRF2), IRF3-G (IRF3 and IRF7), IRF4-G (IRF4, IRF8, IRF9, and IRF10), and IRF5-G (IRF5 and IRF6)—are shown in different colors. Corresponding proteins to transcripts explored using qPCR in the current study are marked by "*".

### Endogenous control (normalizer) selection

Transcript expression levels of each gene of interest (GOI) were normalized to expression levels of two endogenous control genes. These endogenous controls were selected from five candidate normalizers [*ribosomal protein lateral stalk subunit p1* (*rplp1*), *ribosomal protein l32* (*rpl32*), *poly(A) binding protein cytoplasmic 1b* (*papbc1b*), *eukaryotic translation initiation factor 3 subunit d* (*eif3d*), *eukaryotic translation elongation factor 1 alpha 2a* (*ef1a2a*)]. Briefly, the fluorescence threshold cycle (C_T_) values for all 18 samples were measured for each of these transcripts using cDNA representing 5 ng of input total RNA and then analyzed using geNorm (qBase plus, Biogazelle NV, Zwijnaarde, Belgium) (56). This analysis identified *rpl32* and *ef1a2a* as the most stably expressed normalizers, with geNorm M-values of 0.20 and 0.21, respectively.

### Experimental qPCR and data analyses

**Figure 4:**
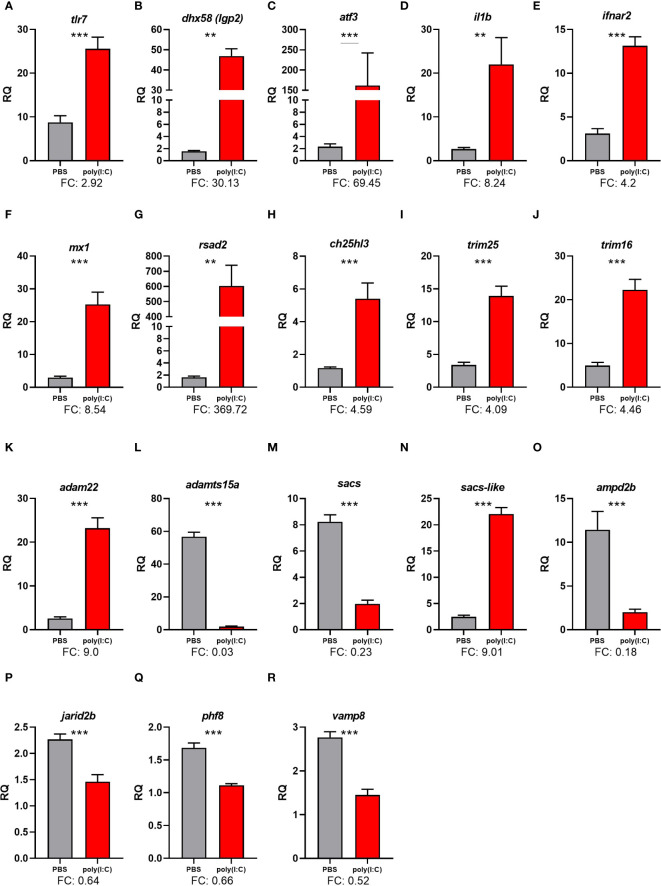
qPCR results of selected transcripts that were identified as differentially expressed in response to polyinosinic:polycytidylic acid [poly(I:C)] in RNA-Seq analyses. Transcript levels are presented as mean ± SE relative quantity (RQ) values (i.e., values for the transcript of interest were normalized to both *rpl32* and *ef1a2a* transcript levels and were calibrated to the individual with the lowest normalized expression level of that given transcript). For transcripts exhibiting homogeneity of variance across samples, significance was assessed using t-tests and is denoted with asterisks. For transcripts with unequal variance across samples, significance was assessed using the Mann–Whitney U test and is denoted with underlined asterisks. For both methods, significance levels are “**” for p ≤ 0.01, and “***” for p ≤ 0.001. FC, fold-change [mean RQ values for poly(I:C)/mean RQ values for phosphate-buffered saline (PBS)]. The plotted transcripts represent **(A)** *Toll-like receptor 7 (tlr7)*, **(B)** *ATP-dependent RNA helicase lgp2* (*dhx58*), **(C)** *activating transcription factor 3* (*atf3*), **(D)** *Interleukin-1 beta-like* (*il1b*), **(E)** *Interferon alpha/beta receptor 2-like* (*ifnar2*), **(F)** *Interferon-induced GTPbinding protein Mx-like* (*mx1*), **(G)** *Radical S-adenosyl methionine domain containing 2* (*rsad2*), **(H)** *Cholesterol 25-hydroxylase like 3* (*ch25hl3*), *E3 ubiquitin/ISG15 ligase*, **(I)** *TRIM25-like* (*trim25*), **(J)** *Tripartite motif-containing protein 16-like* (*trim16*), **(K)** *ADAM metallopeptidase domain 22* (*adam22*), **(L)** *ADAM metallopeptidase with thrombospondin type 1 motif, 15a* (*adamts15a*), **(M)** *Sacsin* (*sacs*), **(N)** *Sacsin-like* (*sacs-like*), **(O)** *Adenosine monophosphate deaminase 2b* (*ampd2b*), **(P)** *Jumonji, AT rich interactive domain 2b* (*jarid2b*), **(Q)** *PHD finger protein 8* (*phf8*), **(R)** *Vesicle-associated membrane protein 8* (*endobrevin*), vamp8.

**Figure 5:**
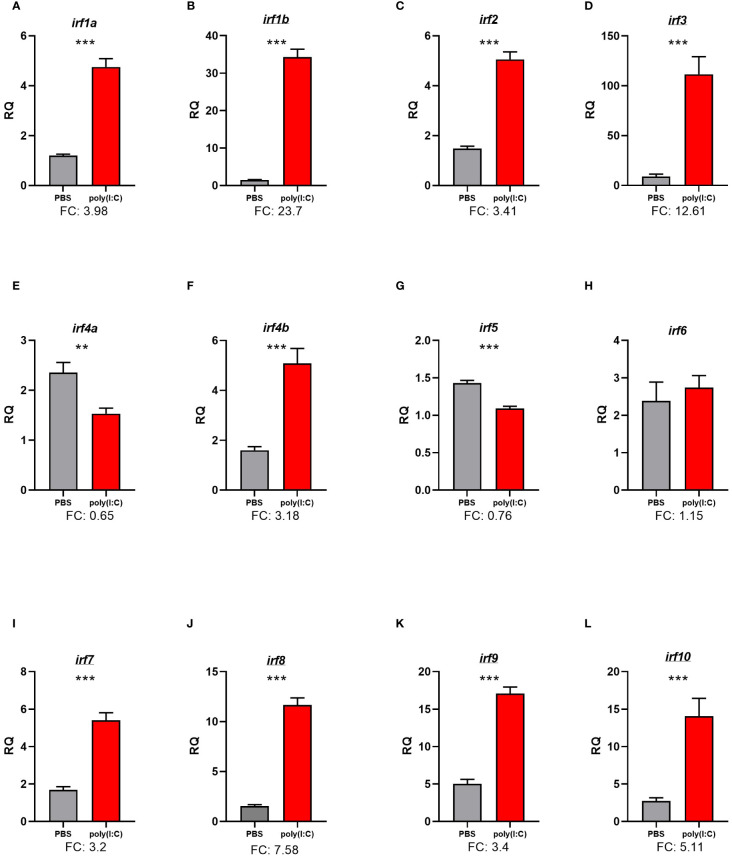
qPCR analysis of the response of the 12 *irf* family members in lumpfish to intraperitoneal (IP) challenge with polyinosinic:polycytidylic acid [poly(I:C)]. Transcript levels are presented as mean ± SE relative quantity (RQ) values (i.e., values for the transcript of interest were normalized to both *rpl32* and *ef1a2a* transcript levels and were calibrated to the individual with the lowest normalized expression level of that given transcript). Significance was assessed using t-tests and is denoted with asterisks (“**” for p ≤ 0.01, and “***” for p ≤ 0.001). FC, fold-change [mean RQ values for poly(I:C)/mean RQ values for phosphate-buffered saline (PBS)]. The *irf*s with underlined gene symbols were identified as differentially expressed in the RNA-Seq analysis and were validated using qPCR. The *irf*s with non-underlined symbols were not differentially expressed in the RNA-Seq analysis using the preidentified cutoff criteria. Both *irf1a* [named in National Center for Biotechnology Information (NCBI) as *irf1-like*] and *irf4b* (named in NCBI as *irf4-like*) were named based on the closest orthologues in the phylogenetic tree (**Figure 3**). **(A)** *interferon regulatory factor 1a (irf1a)*, **(B)** *Interferon regulatory factor 1b (irf1b)*, **(C)** *Interferon regulatory factor 2 (irf2)*, **(D)** *Interferon regulatory factor 3 (irf3)*, **(E)** *Interferon regulatory factor 4a (irf4a)*, **(F)** Interferon regulatory factor 4b (irf4a), **(G)** *Interferon regulatory factor 5 (irf5)*, **(H)** *Interferon regulatory factor 6 (irf6)*, **(I)** *Interferon regulatory factor 7 (irf7)*, **(J)** *Interferon regulatory factor 8 (irf8)*, **(K)** *Interferon regulatory factor 9 (irf9)*, **(L)** *interferon regulatory factor 10. (irf10)*.

The qPCR analyses were conducted according to MIQE guidelines (57). cDNA representing 5 ng of input total RNA was used as a template in the PCRs. The relative quantity (RQ) of each GOI in each of the 18 samples was then determined using the qBase relative quantification framework (58, 59). This was performed using the C_T_ values measured for each GOI, with normalization to both *rpl32* and *ef1a2a* and with the amplification efficiencies incorporated. For each GOI, the sample with the lowest normalized expression was used as the internal calibrator (i.e., assigned an RQ value = 1.0). The RQ values are presented as mean ± SE (**Figures 4**, **5**).

### Phylogenetic tree analysis of putative IRF orthologues

Putative orthologous amino acid (AA) sequences for IRF family members from a fish representing each of the four teleost superorders, namely, Protacanthopterygii (Atlantic salmon), Acanthopterygii (lumpfish), Paracanthopterygii (Atlantic cod), and Ostariophysi (zebrafish; *D. rerio*), were collected from the NCBI GenBank non-redundant (nr) protein database (59 in total). The GenBank accession numbers and AA sequences are provided in **Supplementary Table S5**. The sequences were subjected to BLASTP analyses to help identify all of the IRF isoforms/paralogues for each species and to ensure that all sequences used in the tree were unique (i.e., the tree did not include transcript variants; if present, the best representative sequence was selected). Phylogenetic and molecular evolutionary analyses were conducted using MEGA 11 (v.11.0.13) (60). Briefly, a multiple sequence alignment was performed using the ClustalW algorithm. The phylogenetic tree was then constructed using the Neighbor-Joining method with the Poisson correction; the bootstrap test of phylogeny was performed with 10,000 replicates.

### Statistical analysis

All of the residuals were tested for homoscedasticity and normality (i.e., Levene’s and Shapiro–Wilk tests). Significant (p < 0.05) differences in transcript expression levels between the PBS- and poly(I:C)-injected groups were assessed using either Student’s t-test or the Mann–Whitney U test (for genes that failed the normality test). These analyses were performed using SPSS (IBM SPSS Statistics, Version 25, Armonk, NY, USA). PCA (**Figure 1C**) was performed using PRIMER 7 (PRIMER-E Ltd., Auckland, New Zealand). The scatter plot for the LFC (**Supplementary Figure S2**) was generated using the function “ggscatter” for the library “ggpubr”.

## Results

### Lumpfish head kidney transcriptome assemblies and RNA-Seq analyses

#### Transcriptome sequencing and assemblies

In this study, RNA-Seq was used to profile the responses of lumpfish to viral mimic, poly(I:C). **Supplementary Table S2** summarizes the RNA-Seq read quality control for the 10 samples. The average number of raw reads across all samples was 79.7 M (range, ~62M to 92M). On average, 98% of the read pairs (across all samples) survived the trimming process (range, 97.8% to 98.2%). The average percentage of reads that dropped during trimming was 0.4% (range, 0.3% to 0.5%). Overall, the results show that most reads were successfully trimmed and kept. Both uniquely and multi-mapped reads were used for transcript assembly. Overall, ~94%–96% of the processed reads in each sample were mapped to the lumpfish genome (**Supplementary Table S2**).

#### Poly(I:C)-responsive transcripts in lumpfish head kidney

**Figure 6:**
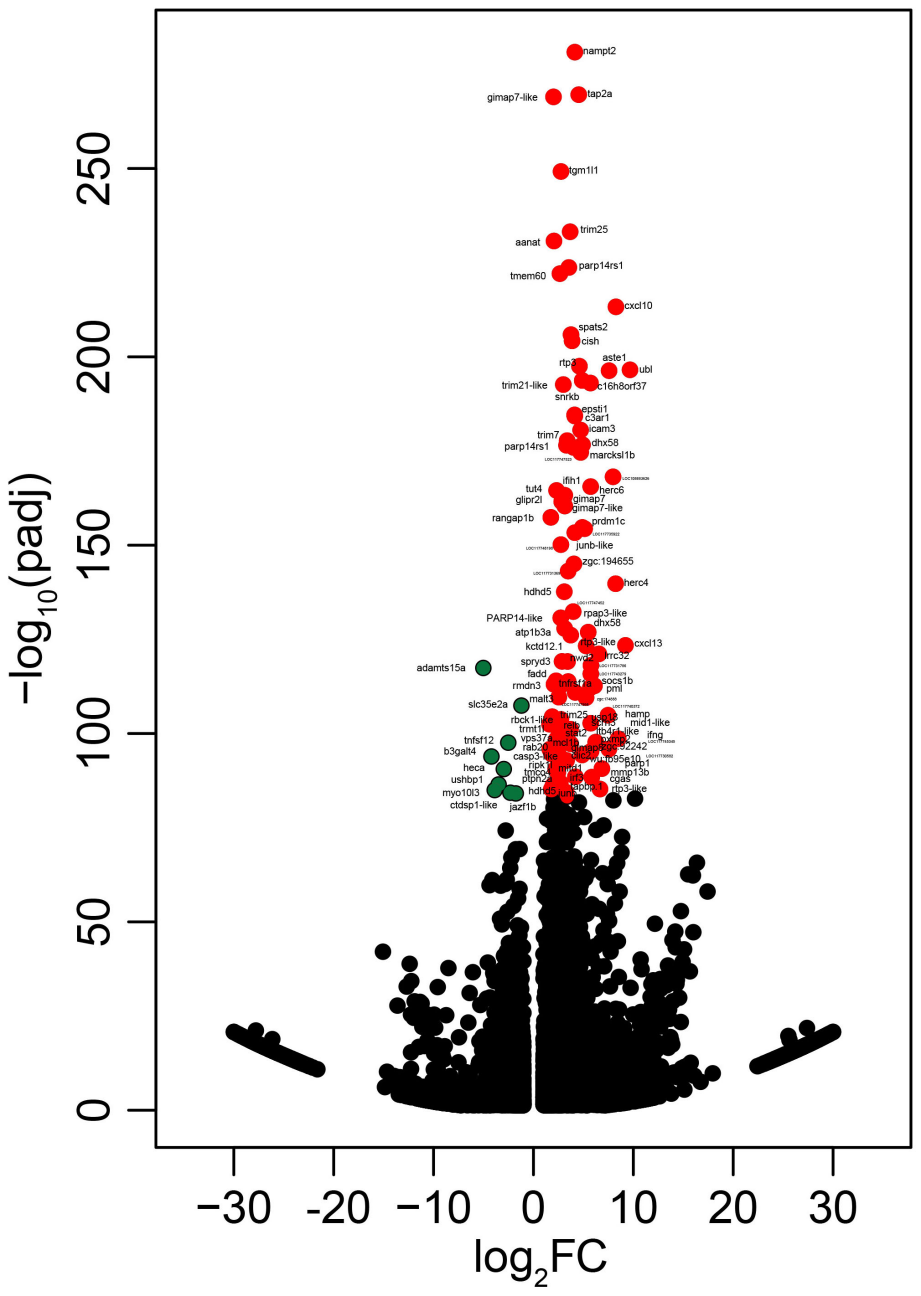
Volcano plot reporting −log_10_ adjusted p-values (padj) against log_2_ fold-changes. Dots represent differentially expressed transcripts. The colored dots represent the most significant transcripts from the upregulated and downregulated probes (red and green, respectively) (**Supplementary Table S3**). Dots are labeled with the gene symbols of the putative human orthologues, and one uncharacterized is labeled with the locus annotation.

We identified 4,499 upregulated and 3,952 downregulated transcripts by poly(I:C) in the head kidney of lumpfish (**Supplementary Table S3**, **Figures 1B**, **6**). All of the samples belonging to a given group (i.e., PBS- or poly(I:C)-injected) clustered together based on the expression of all of the identified DETs. In addition, the PBS- and poly(I:C)-injected fish were clearly segregated in the PCA space. PC1 explained 38.7% of the variability, and PC2 explained 10.4% of the variability (**Figure 1C**).

There were 311 GO terms enriched in the poly(I:C)-induced transcript list including 248 biological processes, five cellular components, and seven molecular functions. The most enriched biological process GO terms were related to “defense response”, “cytokine-mediated signaling pathway”, and “response to other organisms” (**Figure 2**). Enriched molecular function GO terms included “immune receptor activity”, “cytokine receptor binding”, and “interleukin-1 binding”. Enriched cellular component GO terms included “chromosome”, “nuclear protein-containing complex”, and “lipid droplet” (**Supplementary Table S4**). GO terms with the highest percentage of DETs included “regulation of retinoic acid receptor signaling pathway” (**Supplementary Figure S1**).

**Figure 7:**
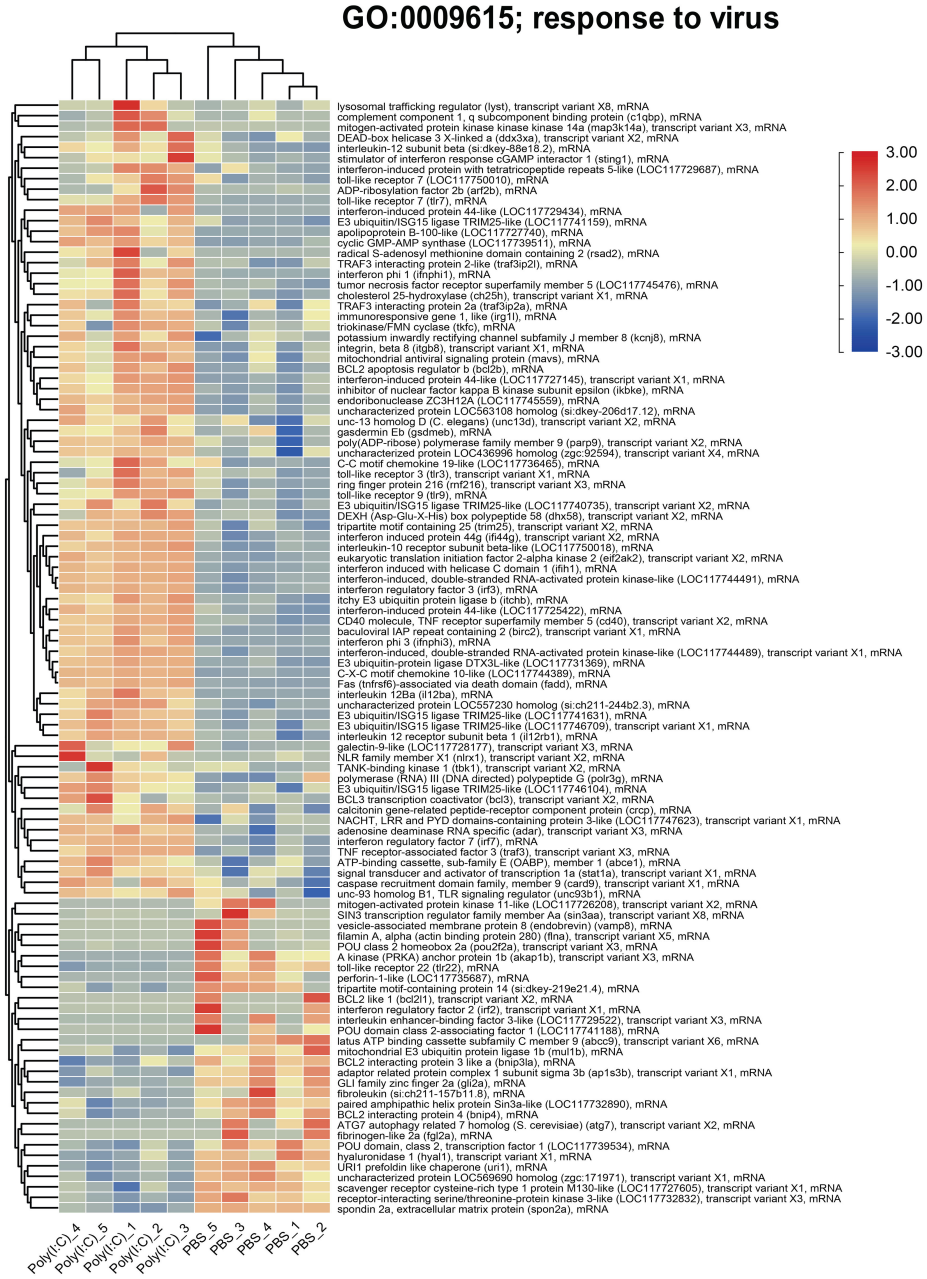
Heatmap and hierarchical clustering of differentially expressed transcripts (DETs) associated with “response to virus” using transcripts per million (TPMs; log_2_ transformed).

The TPMs of the genes represented by the enriched GO term “response to virus” were used to generate the heatmap plotted in **Figure 7**. It showed upregulation of transcripts including *lysosomal trafficking regulator* (*lyst*), *complement component 1*, *q subcomponent binding protein* (*c1qbp*), *DEAD-box helicase 3 X-linked a* (*ddx3xa*), *interleukin-12 subunit beta* (*il12b*), *stimulator of interferon response cGAMP Interactor 1* (*sting1*), *toll-like receptor* 7 (*tlr7*), *cholesterol 25-hydroxylase A* (*ch25ha*), *mitochondrial antiviral signaling protein* (*mavs*), *BCL2 apoptosis regulator B* (*bcl2b*), and *irf2* with poly(I:C) challenge. Also, it showed downregulation of several genes, for example, *vesicle-associated membrane protein 8* (*vamp8*), *spondin2a*, and *scavenger receptor cysteine-rich type 1 protein M130-like.*

**Figure 8:**
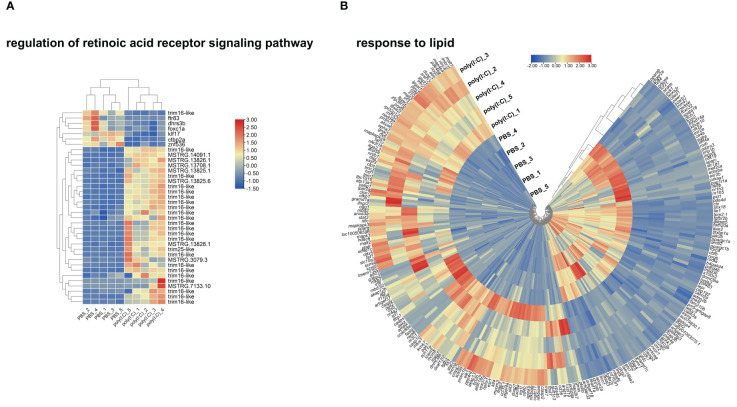
Heatmap and hierarchical clustering of differentially expressed transcripts (DETs) that participated in enriching “regulation of retinoic acid receptor signaling pathway” in panel **(A)** and “response to lipid” in panel **(B)** using transcripts per million (TPM; log_2_ transformed).

To investigate dysregulation in metabolism during a viral mimic challenge, we plotted a heatmap for DETs representing the enriched GO terms “regulation of retinoic acid receptor signaling pathway” and “response to lipid” GO terms (**Figures 8A, B**). The GO term regulation of the retinoic acid receptor signaling pathway was one of the GO terms with the highest percentage of DETs. **Figure 8A** shows the upregulation of different transcripts including *tripartite motif containing 16-like* (*trim16-like*) and *tripartite motif containing 25-like* (*trim25-like*). Also, it shows downregulation of genes including *dehydrogenase/reductase 3B* (*dhrs3b*), *kruppel-like factor 17* (*klf17*), *c-terminal binding protein 2a* (*ctbp2a*), and others representing *mitogen-activated protein kinase 11-like*, *vesicle-associated membrane protein 8* and *fibroleukin* with poly(I:C) injection.

The GO term “response to lipid” was enriched with a mixture of upregulated and downregulated genes (**Figure 8B**). For example, *mucosa-associated lymphoid tissue lymphoma translocation protein 1* (*malt1*), *NLR family CARD domain containing 3-like* (*nlrc3l*), and *B-cell lymphoma 2 B* (*bcl2b*) were upregulated with poly(I:C). While *frizzled class receptor 4* (*fzd4*), *annexin a2 receptor* (*anxa2r*), *prostaglandin E receptor 2* (*ptger2a*), *peroxisome proliferator-activated receptor alpha B* (*pparab*), and *peroxisome proliferator-activated receptor gamma coactivator 1 alpha* (*ppargc1a*) were downregulated with poly(I:C) stimulation.

### qPCR validation

The qPCR results confirmed the RNA-Seq results for all of the selected transcripts (**Supplementary Figure S2**). There was a significant correlation (*p* = 0.007; Pearson’s coefficient = 0.61) between the qPCR and the RNA-Seq results (**Supplementary Figure S2**).

The qPCR analysis confirmed the poly(I:C) induction of *tlr7*, *ATP-dependent RNA helicase lgp2* (*dhx58*), *activating transcription factor 3* (*atf3*), *interleukin 1 beta* (*il1b*), *interferon alpha/beta receptor 2* (*ifnar2*), *interferon-induced GTP-binding protein Mx-like* (*mx1*), *radical S-adenosyl methionine domain containing 2* (*rsad2*), *ch25ha*, *trim25*, *trim16*, *ADAM metallopeptidase domain 22* (*adam22*), and *spastic ataxia of Charlevoix-Saguenay* (*sacsin*)*-like* (*sacs-like*) (**Figure 4**).

The qPCR analysis confirmed the poly(I:C) repression of *ADAM metallopeptidase with thrombospondin type 1 motif 15* (*adamts15a*), *sacs*, *adenosine monophosphate deaminase 2b* (*ampd2b*), *jumonji and at-rich interaction domain containing 2* (*jarid2b*), *histone lysine demethylase phf8* (*phf8*), and *vamp8* (**Figure 4**).

### Lumpfish *irf* family qPCR and phylogenetic analysis

To characterize the response of the 12 *irf* family members in lumpfish stimulated with poly(I:C), their transcript expression levels were assessed using qPCR. Seven members, i.e., *irf1b*, *irf2*, *irf3*, *irf7*, *irf8*, *irf9*, and *irf10*, were identified as dysregulated with poly(I:C) challenge in the RNA-Seq analyses, whereas *irf1a*, *irf4a*, *irf4b*, *irf5*, and *irf6* were not detected as differentially expressed using a cutoff level of LFC = 1.0. With the exception of *irf6*, all *irf* family members subjected to qPCR analysis were found to be poly(I:C)-responsive. The transcript levels of *irf1a*, *irf1b*, *irf2*, *irf3*, *irf7*, *irf8*, *irf9*, and *irf10* were upregulated with poly(I:C) injection when compared with the PBS-injected group. The levels of *irf4a* and *irf5* were suppressed in response to poly(I:C) injection. The levels of *irf6* were not significantly different between the poly(I:C)- and PBS-injected groups (**Figure 5**).

A total of 59 IRF family members in species representing the four teleost superorders (lumpfish, Acanthopterygii; Atlantic salmon, Protacanthopterygii; zebrafish, Ostariophysi; and Atlantic cod Paracanthopterygii), identified in the GenBank nr protein database, were used to build a phylogenetic tree (**Figure 3**). As anticipated, the teleost IRF sequences cluster into the four previously defined subgroups: IRF1-G (IRF1 and IRF2), IRF3-G (IRF3 and IRF7), IRF4-G (IRF4, IRF8, IRF9, and IRF10), and IRF5-G (IRF5 and IRF6) (61). The phylogenetic tree shows that lumpfish IRF2, IRF4a, IRF4b, IRF5, IRF6, IRF7, IRF8, and IRF10 are evolutionarily more closely related to the Atlantic cod compared with the Atlantic salmon or zebrafish putative orthologues (**Figure 3**). However, the tree also reveals that lumpfish IRF3 is most closely related to Atlantic salmon IRF3 (i.e., sharing a branch point); likewise, lumpfish IRF9 is more closely related to Atlantic salmon IRF9 paralogues (IRF9–1 and IRF9–2, arising from duplication in the salmonid lineage) than to Atlantic cod or zebrafish IRF9 sequences (**Figure 3**). Finally, for IRF1a and IRF1b, zebrafish and Atlantic salmon sequences are more closely related to each other than either are to the lumpfish putative orthologous sequences.

## Discussion

The current RNA-Seq results detected an extensive global gene expression response (i.e., 4,499 upregulated and 3,952 downregulated DETs) in the head kidney of poly(I:C)-challenged lumpfish, shedding light on the molecular mechanisms and immune pathways involved in response to this viral mimic. GO term analyses identified enriched immune-related GO terms consistent with an immune response to a viral challenge based on the current knowledge about molecular antiviral responses in teleost fish (13, 14). Several leading immune-related GO terms (e.g., “antigen processing and presentation of peptide antigen via MHC class I”, “leukocyte apoptotic process”, “pattern recognition receptor signaling pathway”, “response to virus”, “type I interferon signaling pathway”, and “NIK/NF-kappaB signaling”) were primarily represented by poly(I:C)-induced DETs compared with downregulated ones (**Figure 2B**). Many of the leading GO terms within cellular component and biological process categories (e.g., “RNA splicing, via transesterification reactions with bulged adenosine as nucleophile”, “chromosome”, “nuclear protein-containing complex”, and “cell migration”) were represented by a higher number of downregulated DETs compared with upregulated DETs. These results suggest a possible shift in cellular activity toward fighting infection, highlighting gene expression regulation patterns toward the induction of immune-relevant genes.

Our GO term enrichment results generally agreed with those of a recent *in vitro* study (32) investigating the effects of poly(I:C) on the transcriptome of lumpfish primary leukocytes after 24 h of exposure, especially in terms of general immune-relevant GO terms such as “cytokine receptor binding” and “response to virus”. However, the present study identified a more varied suite of enriched biological processes, including GO terms related to cell migration (e.g., “leukocyte migration”), apoptosis (e.g., “leukocyte apoptotic process”), and adaptive immunity (e.g., “antigen processing and presentation of peptide antigen via MHC class I”), which may be a consequence of analyzing transcriptome changes in head kidney samples as opposed to primary leukocytes. Notably, in Rao et al. (32), biological processes associated with the metabolism of nitrogen-containing compounds (e.g., “peptide metabolic process”) were dominant. Our analyses also identified enriched GO terms related to retinoic acid (e.g., “regulation of retinoic acid receptor signaling pathway”) like in Rao et al. (32) but also other terms associated with lipids (e.g., “lipid droplet” and “response to lipid”). We anticipate that single-cell RNA-Seq in lumpfish will likely allow the assignment of transcript expression changes to specific cells within the head kidney, elucidating GO terms enriched in each type of cell.

During viral infection, the host’s metabolism undergoes various changes to combat the virus and support the immune response. This may be evidenced in the current study by enrichment in GO terms relevant to metabolism (e.g., GO terms related to lipid and retinoic acid metabolism; **Supplementary Table S3**, **Figures 2A, B**). In mammals, lipid droplets (62, 63) and retinoic acid (64) are key players in the viral infection mechanisms and the inflammatory processes they trigger. The GO term “regulation of retinoic acid receptor signaling pathway” was one of the GO terms with the highest percentage of DETs (**Supplementary Figure S1**). The heatmap for “regulation of retinoic acid receptor signaling pathway” (**Figure 8A**) shows that several *trim16-like* transcripts were upregulated by poly(I:C), except for one downregulated transcript (accession number: XM_034555257.1). The heatmap also showed decreased transcript levels for *ftr83* (*tripartite motif-containing protein 11*), *dhrs3b*, *foxc1a* (*forkhead box C1a*), *klf17* (*krüppel-like factor 17*), *ctbp2a* (*C-terminal binding protein 2a*), and *znf536* (*zinc finger protein 536*) in the poly(I:C)-injected fish (**Figure 8A**). These poly(I:C)-responsive genes encode proteins involved in regulating the retinoid metabolic process (*dhrs3b*), regulation of cell proliferation (*foxc1a*), differentiation (*klf17*), and response to external stress (e.g., *ctbp2a* and *znf536*) (65–68). Their regulation may be instrumental to mounting the immune response to the viral mimic challenge in lumpfish and highlights the role of vitamin A (retinoic acid) during viral infection in lumpfish. However, vitamin A modulation of the immune response during viral infection requires further research.

Several upregulated and downregulated genes by poly(I:C) identified herein contributed to enriching the GO term “response to lipid” (**Figure 8B**). For example, transcripts encoding receptors (e.g., *fzd4*, *anxa2r*, and *ptger2a*) and transcription factors involved in metabolism, energy homeostasis, and immunomodulation (69) (e.g., *pparab* and *ppargc1a*) were found downregulated by the viral mimic challenge (**Figure 8B**). Others were found to be upregulated, for example, transcripts encoding proteins involved in the activation of the transcription factor NF-κB (i.e., *malt1*), intracellular pattern recognition receptors previously suggested to regulated innate immune response (70) (e.g., *nlrc3l*), and apoptosis (e.g., *bcl2b*) (71). Overall, the enrichment of “response to lipid” and other lipid-related GO terms may highlight the role of lipids during viral infection (62, 72–74), emphasizing the potential of dietary lipids to modulate the antiviral fish immune response.

The enriched GO term “response to virus” was predominantly represented by upregulated transcripts involved in antiviral immune defense [e.g., *mavs*, and *signal transducer and activator of transcription 1a* (*stat1a*)] (75), inflammation (e.g., *ch25h*, *tumor necrosis factor-alpha*, and *il6*) (76), oxidative stress (e.g., *c1qbp*) (77), and apoptosis regulation (e.g., *bcl2b*) (78). Although to a lesser extent, this GO term was also represented by poly(I:C)-repressed genes related to various cellular process, like vesicle trafficking (e.g., *vamp8*) (79) and autophagy (e.g., *autophagy related 7 homolog* and *atg7*) (80) as well as deubiquitination and protein metabolism (e.g., *mitochondrial E3 ubiquitin protein ligase 1b* and *mul1b*) (81). The dysregulated transcripts representing this GO term indicate the involvement of genes with diverse functions in the antiviral immune response.

### qPCR results of lumpfish response to poly(I:C)

In the current study, all of the 18 genes used in the qPCR validated the RNA-Seq, indicating the reliability of the RNA-Seq results (**Supplementary Figure S1**). In the qPCR study, two pattern recognition receptor (PRR)-encoding genes, *tlr7* and *dhx58*, were found to be upregulated by poly(I:C). *Tlr7* contributed to 59 enriched GO terms, including “NIK/NF-kappaB signaling”, “pattern recognition receptor signaling pathway”, “response to virus”, and “regulation of cytokine production”. Similarly, *dhx58* contributed to enriching 83 GO terms, including “response to virus”, “positive regulation of intracellular signal transduction”, and “regulation of response to stimulus” (**Supplementary Table S3**). PRRs detect conserved pathogen-associated molecular patterns (PAMPs) and consequently activate the innate immune response (82). Upon recognition of viral single-stranded RNA molecules, TLR7 triggers the production of type I IFNs and pro-inflammatory cytokines, which are critical for the host’s antiviral response (83–85). DHX58 is a member of the RLR family and an ATP-dependent RNA helicase, also known as LGP2 or RIG-I-like receptor 1 (RLR1), and has several antiviral roles such as regulation of TLRs and RLRs (86). *Dhx58* was also found to be upregulated in Atlantic cod spleen and brain after IP injection with poly(I:C) in the brains of nodavirus-positive Atlantic cod (87, 88) and lumpfish infected with *R. salmoninarum* (2). *Dhx58* was upregulated in lumpfish larvae after oral immunization against *Vibrio anguillarum* (8); also, it was highly upregulated in lumpfish leukocytes after stimulation with poly(I:C) (32). The results of previous and current studies suggest that DHX58 plays a role in both the antibacterial and antiviral immune responses of lumpfish, whereas TLR7 dysregulation occurs following the activation of its antiviral responses.

In the current study, the transcript levels of *atf3* were strongly upregulated (over 69-fold) in the head kidneys of lumpfish stimulated with poly(I:C), as it was previously reported for Atlantic salmon macrophages (13) and Atlantic cod spleen (89). Mammalian ATF3 is activated by cellular stress response pathways and plays a role in the host’s immune response to viral infections (89, 90). ATF3 upregulation during viral infections promotes a stronger immune response and increased resistance in various mammalian species including mice and humans (91–93). Additionally, human ATF3 can directly inhibit the replication of some viruses by suppressing their transcription (93) and regulating the expression of the host’s immune-related genes such as IFN-induced and pro-inflammatory cytokines (94). While our results indicate the involvement of *atf3* in lumpfish’s antiviral immune response, its viral inhibitory and regulatory functions are yet to be investigated in this species.

Two lumpfish transcripts encoding proteins classified as cytokines and cytokine receptors (i.e., *il1b* and *ifnar2*) were found to be over fourfold upregulated by poly(I:C). Several ILs contributed to enriching key GO terms, including “response to interleukin-1”, “signaling receptor binding”, “leukocyte migration”, and “defense response to other organism” (**Supplementary Table S3**). IL1B is a pro-inflammatory cytokine mediating the immune response of fish to viral and bacterial infection (95, 96). The production of IL1B by immune cells such as macrophages is triggered by the detection of viral nucleic acid by PRRs, such as TLRs (97). Previous studies showed *il1b* induction in the kidney of Sockeye salmon (*Oncorhynchus nerka*) infected with infectious hematopoietic necrosis virus (IHNV) (95, 96). Type I interferons, including IFNA, are produced following viral detection (98) and can activate cellular antiviral immune mechanisms (e.g., the expression of IFN-stimulated genes) and the recruitment of immune cells such as natural killer cells and T cells (98). It was previously reported that IFNA inhibited Salmonid Alphavirus Subtype 3 replication in a salmon cell line (i.e., TO cells originated from head kidney leukocytes) (99). Additionally, *ifna* was found upregulated in the head kidney of Atlantic salmon with New Piscine Orthomyxovirus (POMV) infection (100). These results collectively emphasize the conserved roles of IL1B and IFNA in the antiviral responses of lumpfish as in other teleost fishes.

Antiviral markers *mx1*, *rsad2* (alias *viperin*), and *ch25hl3* were found upregulated by poly(I:C) stimulation. *Rsad2* and *ch25hl3* contributed to enriching several GO terms, e.g., “response to virus” (**Figure 7**) and “defense response to other organism” (**Supplementary Table S3**). Atlantic salmon *mx* and *rsad2* showed strong upregulation in ISAV-infected TO cells (101), poly(I:C)-stimulated macrophages (13), and the head kidney of poly(I:C)-injected fish (55). MX1 plays a role in the salmon immune response to viral infections, notably myxoviruses such as ISAV (102), and spring viraemia of carp virus (SVCV) (103). The poly(I:C)-induced *mx1* can enhance resistance to viral infections in salmonids (104). RLR-activated RSAD2 (105) regulates the RLR signaling pathway through phosphorylation of downstream targets, e.g., MAVS (mitochondrial antiviral-signaling protein) and IRF3 [interferon regulatory factor 3; one of the top upregulated transcripts in the current study], leading to amplified antiviral response (106). CH25H plays a role in the immune response of salmon to bacterial infections (e.g., *Renibacterium salmoninarum* and *Piscirickettsia salmonis*) (49, 107, 108). Viral infections can lead to increased expression of *ch25h* in salmon (109). Additionally, it has been found that fish CH25H can directly inhibit the replication of some viruses (110). Altogether, *mx1*, *rsad2*, and *ch25hl3* responses seen herein reflect the activation of antiviral agents by poly(I:C) in lumpfish and suggest these transcripts as potential antiviral biomarkers for this species.

The mRNA levels of *trim16* and *trim25*, which play roles as immune regulators, were upregulated by poly(I:C) in lumpfish. TRIM25 shared in enriching several GO terms, such as “signaling receptor binding”, “response to virus”, “regulation of cytokine production”, and “response to lipid” (**Supplementary Table S3**). TRIM16 and TRIM25 are E3 ubiquitin ligases that play key roles in the host’s immune response to viral infections (111). Lumpfish leukocytes stimulated with poly(I:C) showed higher levels of *trim25* from 6 to 24 h post-challenge (32). In Atlantic salmon TO cells, *trim16* and *trim25* were strongly upregulated by ISAV infection (101). The literature regards TRIM16L as a negative regulator of IFN-mediated antiviral responses in fish. However, the role of this protein in antiviral immune responses may be cell type-dependent, based on the available gene expression regulation data from fish and human cells exposed to viral infection (65, 112, 113). Human TRIM25 is reported to be able to target and degrade viral proteins (e.g., influenza A virus), thereby inhibiting viral replication (114). The conserved induction of *trim16* and *trim25* found in the current study highlights the importance of these factors in the antiviral response of lumpfish and suggests their role in the regulatory mechanism by which lumpfish respond to viral pathogens.

*Adam22* was found upregulated with poly(I:C) stimulation and contributed to enriching “organonitrogen compound metabolic process” GO term (**Supplementary Table S3**). In a similar direction to our results, transcript levels of *adam22* were previously found upregulated in the brain of Atlantic cod injected with poly(I:C) (88). Human ADAM22 has been shown to mediate the entry of the human rhinovirus (HRV) (115). However, the role of ADAM22 during antiviral immune response remains to be elucidated in lumpfish.

Lumpfish *sacs-like* (accession number: XM_034562115) was upregulated by poly(I:C) stimulation, whereas its paralogue (XM_034549198) was poly(I:C)-suppressed (**Figure 4**, **Supplementary Table S3**). The levels of Atlantic cod *sacs* have been reported to increase in the brain of nodavirus carrier fish (88) and Atlantic cod macrophages stimulated with poly(I:C) (14). It was also found upregulated in the brain of sockeye salmon infected with IHNV (116). The current study results may indicate paralogue-specific functions for lumpfish *sacs* genes. Opposite transcriptional regulation was previously reported for some paralogues in zebrafish and salmon (117, 118). However, further research is needed to understand the implications of the different *sacs* regulation patterns in lumpfish antiviral immune responses.

We identified several immune-related genes downregulated in poly(I:C)-stimulated lumpfish. The transcript levels of *ampd2b* were found to be downregulated by poly(I:C) and contributed to enriching the “GTP metabolic process” GO term. AMPD2 is an enzyme involved in the regulation of cellular energy levels, i.e., purine metabolism by converting adenosine monophosphate (AMP) to inosine monophosphate (IMP) (119). Therefore, the downregulation of *ampd2b* may indicate a decrease in energy production or utilization in the head kidney cells of the lumpfish, which could be part of the response to the stress caused by the viral mimic. Transcripts encoding proteins that are involved in epigenetic regulation and histone modification, such as *jarid2b* (120) and *phf8* (121), were suppressed (qPCR and RNA-Seq results) in the head kidney of the poly(I:C)-injected fish (**Figure 4**, **Supplementary Table S3**). *Jarid2b* contributed to enriching several GO terms, including “regulation of cellular protein metabolic process”, and *phf8* contributed to enriching different GO terms including “immune system development” (**Supplementary Table S4**). Although histone modifications were found to play a protective role in the body’s defense against viral infections (122), the functions of *jarid2b* and *phf8* in epigenetic interaction and immune response of teleost fish require further study. The transcript levels of *vamp8* were downregulated in both the RNA-Seq and qPCR results (**Figure 4**, **Supplementary Table S3**). Also, they shared in enriching several GO terms, e.g., “regulation of cell activation” and “positive regulation of multicellular organismal process” (**Supplementary Table S4**). VAMP8 plays several roles in intracellular membrane trafficking and fusion (123). VAMP8 may also be implicated in the release of cytokine and the inhibition of phagocytosis (124). The downregulation of *vamp8* may be part of the above-hypothesized inhibited cellular function or the immune response regulation.

### Different members of *irf* family response to poly(I:C)

IRFs are a family of transcription factors that play a key role in the host’s immune response to viral infections, especially in regulating IFN and interferon-stimulated genes (125). In the current study, the qPCR results showed that *irf1a*, *irf1b*, *irf2*, *irf3*, *irf4b*, *irf7*, *irf8*, *irf9*, and *irf10* were significantly and strongly upregulated (FC range, 3.2 for *irf4b* and *irf7* to 23.7 for *irf1b*) by poly(I:C) injection (**Figure 5**). Similar to qPCR results, the RNA-Seq also identified significant induction of *irf1b*, *irf2*, *irf3*, *irf7*, *irf8*, *irf9*, and *irf10* by poly(I:C) (**Supplementary Table S3**, **Figure 5**). Various *irf*s (e.g., *irf1*, *irf2*, *irf3*, *irf4b*, *irf7*, *irf9*, and *irf10*) (46) were previously found upregulated with poly(I:C) or viral infection in teleost species (13, 126–129). The *irf* family members (i.e., *irf1*, *irf2*, *irf3*, *irf7*, *irf8*, and *irf10*) detected in our transcriptomic analysis contributed to enriching various GO terms, for example, “pattern recognition receptor signaling pathway”, “response to type I interferon”, “regulation of signaling”, and “cytokine production”. In contrast, *irf9* was only involved in enriching “cytokine production”. These IRFs have been shown to play several roles in the activation of innate immunity and the production of type I interferons, which are important in the defense against viral infections (46, 47). IRF1 is a negative regulator of cytokine-induced cell proliferation in mammals (130). It has been reported that *irf2* positively regulates the antiviral responses of large yellow croaker (*Larimichthys crocea*) (129). IRF7 and IRF3, key family members involved in antiviral responses, are activated downstream of the RLR and RLR pathways and enhance the expression of several immune genes such as IFNs (131). Human IRF8 supports the rapid expansion of virus-specific natural killer cells by enhancing the expression of genes involved in the cell cycle (132). IRF9 mediates the type I interferon responses, resulting in the production of IFN-induced genes (133). Zebrafish IRF 2, 4b, and 10 were suggested to be negative regulators of IFN (134), indicating that their role in the host’s antiviral response may be different among species. The induction of *irf* genes by poly(I:C), alongside dysregulation of several genes involved in TLR, RLR, and IFN pathways seen herein, highlights the importance of these transcription factors in antiviral responses of lumpfish. However, despite conserved structure, IRF family members may have species-specific regulatory functions (135, 136); further studies are needed to functionally characterize the lumpfish IRFs.

Unlike other lumpfish *irf*s studied here, *irf4a* and *irf5* were significantly downregulated (less than twofold) in response to poly(I:C). IRF4 was previously reported to play a role in the differentiation of immune cells and the regulation of the immune response (137–139). In agreement with these findings, seabream *irf5* was found to be downregulated with NNV at 12 h post-infection (140). Poly(I:C)-dependent *irf4a* and *irf5* downregulation seen herein suggest their potential role in the regulation of antiviral responses in lumpfish.

The phylogenetic analysis of IRF sequences from lumpfish, Atlantic salmon, Atlantic cod, and zebrafish was used to examine the evolutionary history of IRF family members in lumpfish in the current study. The majority of lumpfish IRF family members (e.g., IRF2, IRF4a, IRF4b, IRF5, IRF6, IRF7, and IRF10) were grouped with the corresponding Atlantic cod orthologues. Overall, the phylogenetic tree suggests a high degree of similarity and evolutionary conservation between the IRF family members in lumpfish and their orthologues in other species. The observed grouping supports the notion that these specific IRF genes have been conserved over evolutionary time in teleost fishes, highlighting their functional importance across species, e.g., lumpfish and Atlantic cod. This finding contributes to our understanding of the evolutionary relationships and conservation of IRF genes in lumpfish.

## Conclusion

Our findings suggest that poly(I:C) injection dysregulated diverse pathways associated with the antiviral immune system, cellular differentiation, cytokine production and response, NF-κB signaling, response to retinoic acid and lipids, and cell migration in the lumpfish head kidney. The leading GO terms related to cellular processes were enriched with more downregulated transcripts than the upregulated ones (e.g., “chromosome”). In contrast, GO terms with immune-relevant enriched pathways were dominated by upregulated genes. Our qPCR results validated the upregulation of genes involved in innate immunity and antiviral defense mechanisms and the downregulation of those with putative roles in cellular processes (e.g., histone modification: *jarid2b* and *phf8*).

The regulation of several lumpfish *irf* family members with poly(I:C) injections suggests their involvement in the host’s antiviral response. However, the functional characterization of IRF family members in lumpfish requires additional investigation. The results of the current study provide valuable insight into the underlying mechanisms of the induction of the innate immune system using poly(I:C) and suggest potential targets for developing therapeutic strategies and evaluating vaccine efficacy in lumpfish.

## Data Availability

The datasets presented in this study can be found in online repositories. The names of the repository/repositories and accession number(s) can be found below: PRJNA1082277 (SRA).
