# Supplementary figures and images for: Transcriptomic response of lumpfish (Cyclopterus lumpus) head kidney to viral mimic, with a focus on the interferon regulatory factor family
Source: Front Immunol. 2024 Aug 15;15:1439465. doi: 10.3389/fimmu.2024.1439465 (PMC11357929; doi:10.3389/fimmu.2024.1439465)

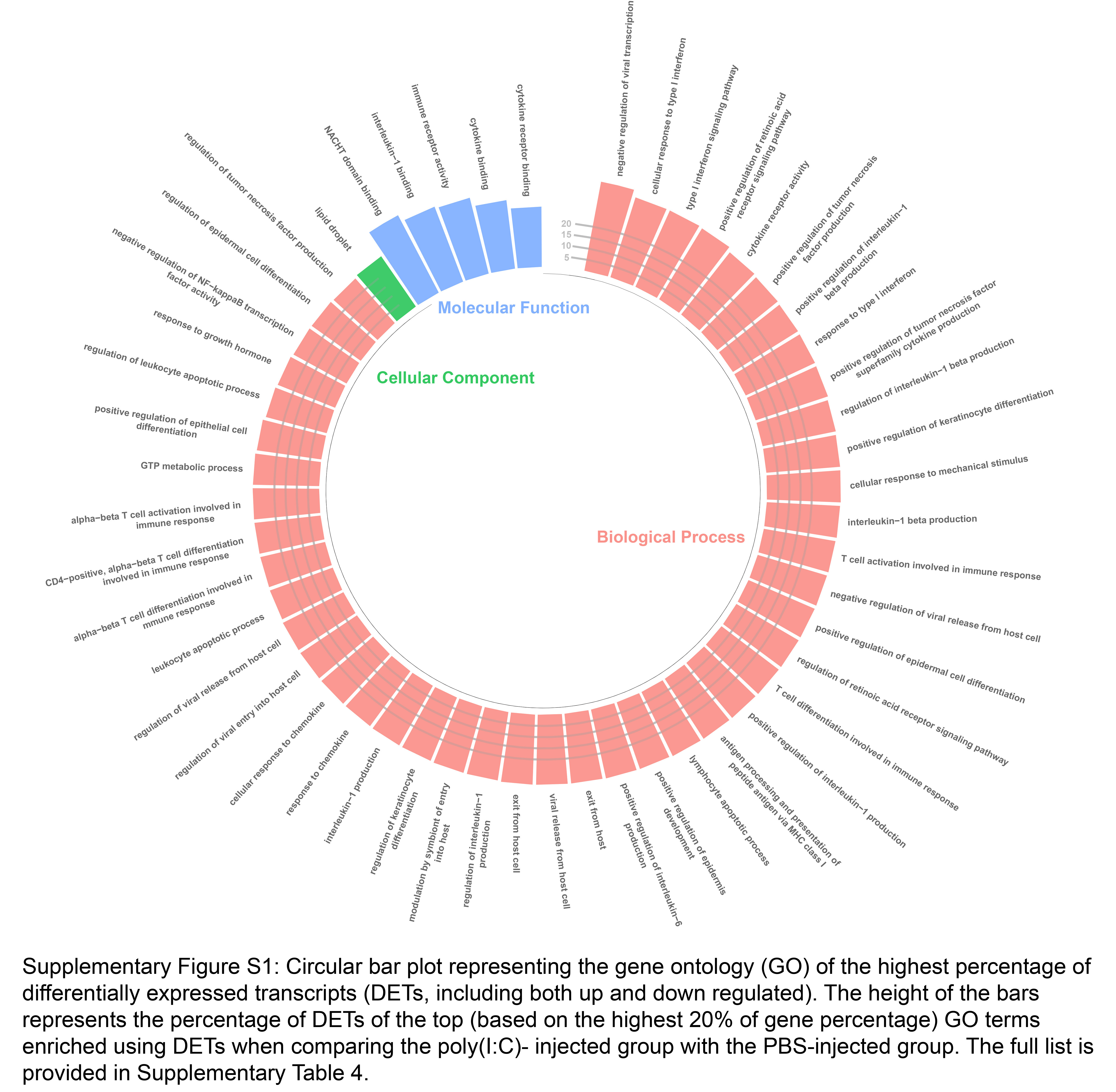

Supplement: Supplementary file 1 [file Image1.jpeg]

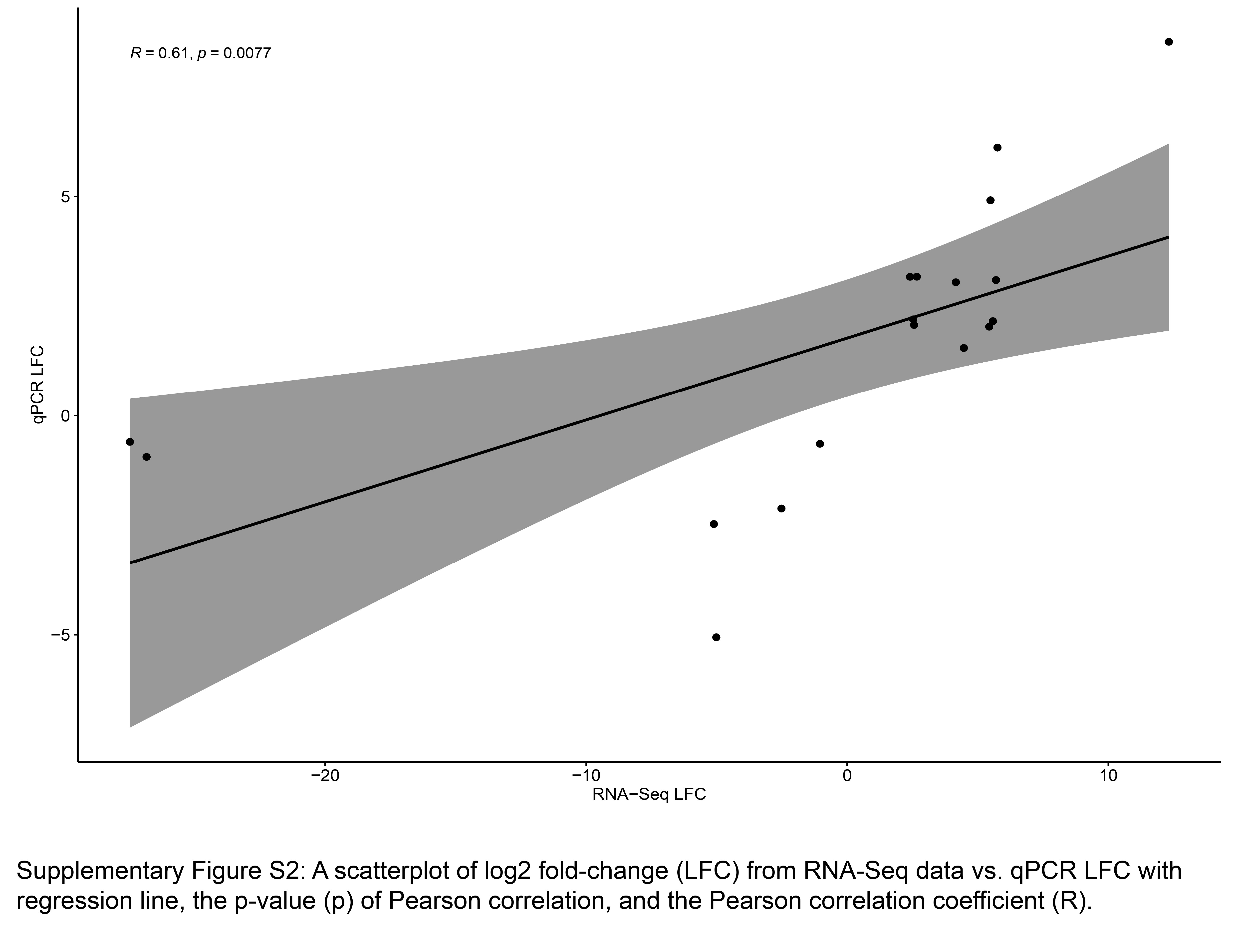

Supplement: Supplementary file 2 [file Image2.jpeg]
