# Supplementary material for: Transcriptomic response of lumpfish (Cyclopterus lumpus) head kidney to viral mimic, with a focus on the interferon regulatory factor family
Source: Front Immunol. 2024 Aug 15;15:1439465. doi: 10.3389/fimmu.2024.1439465 (PMC11357929; doi:10.3389/fimmu.2024.1439465)
Supplement: Supplementary file 3 [file Table1.docx]

**Supplementary Table S1. Examples of RNA viruses that have been detected in *Cyclopterus lumpus***

| **Name of virus** | **Reference and notes** |
| --- | --- |
| **dsRNA viral genome** |  |
| CLuTLV (*Cyclopterus lumpus* toti-like virus) | Similar to family *Totiviridae* (dsRNA genome), detected by sequencing in farmed lumpfish fry after a mortality event (but clinical signs not subsequently associated with CLuTLV). New genus *Pistolvirus* suggested, containing toti-like viruses found in Golden shiner (GSTLV-1), Common carp (CCTLV-1), Atlantic salmon (Piscine myocarditis virus) and CLuTLV^23^. |
| **Positive-sense ssRNA viral genome** |  |
|  |  |
| CLuV (*Cyclopterus lumpus* virus)  NNV (nervous necrosis virus) isolated from asymptomatic wild wrasses in Norway and Sweden (Korsnes et al. 2017)  *Cyclopterus lumpus* coronavirus (CLuCV) | Family *Flaviviridae*: positive-sense ssRNA genome, first identified by sequencing from liver and kidney of a diseased farmed lumpfish^145^. Detected in mixed tissue samples (kidney, spleen, brain, liver) by real-time RT-PCR after a mortality event in farmed lumpfish in England ^24,145^. |
|  | Family *Nodaviridae*, genus *Betanodavirus*: positive-sense ssRNA genome. Lumpfish exposed to NNV from wrasses were shown to be susceptible to *Betanodavirus*, with detected viral RNA suggesting replication in brain and characteristic lesions seen in brain and eye (but no nervous signs)^25^. |
|  |  |
|  | Some reports indicated the infection of lumpfish with coronavirus^146^ |
|  |  |

**Supplementary Table S2** RNA-Seq reads summary for different libraries under PBS and Poly(I:C) Conditions:

| **Library name** | **PBS_1** | **PBS_2** | **PBS_3** | **PBS_4** | **PBS_5** | **Poly(I:C)_1** | **Poly(I:C)_2** | **Poly(I:C)_3** | **Poly(I:C)_4** | **Poly(I:C)_5** |  |
| --- | --- | --- | --- | --- | --- | --- | --- | --- | --- | --- | --- |
| **Raw reads ^a^** | 78858437 | 92971298 | 80045572 | 89870909 | 81996918 | 80496906 | 67816251 | 79824436 | 62047871 | 83878603 |  |
| **Survived reads ^b^** | 77342033 (98.08%) | 91225347 (98.12%) | 78591875 (98.18%) | 88188828 (98.13%) | 80222631 (97.84%) | 78880636 (97.99%) | 66623672 (98.24%) | 78088012 (97.82%) | 60955145 (98.24%) | 82237312 (98.04%) |  |
| **Dropped ^c^** | 318895 (0.40%) | 354617 (0.38%) | 333572 (0.42%) | 374914 (0.42%) | 446976 (0.55%) | Dropped: 359734 (0.45%) | 238212 (0.35%) | 379583 (0.48%) | 189094 (0.30%) | 312400 (0.37%) |  |
|  |  |  |  |  |  |  |  |  |  |  |  |
| **overall alignment rate ^d^** | 95.63% | 95.46% | 95.58% | 94.95% | 95.62% | 95.13% | 95.48% | 95.32% | 95.50% | 95.78% |  |

^a^ Raw reads are the original sequences obtained directly from the sequencing machine

^b^ The survived reads are the sequences that pass this filtering step and are deemed of sufficient quality for further analysis.

^C^ The dropped reads are the raw reads that were removed or discarded during the quality control and filtering process.

^D^ The overall alignment rate indicates the proportion of survived reads that successfully align (or map) to the reference.
